# Clinical Symptoms in Fibromyalgia Are Better Associated to Lipid Peroxidation Levels in Blood Mononuclear Cells Rather than in Plasma

**DOI:** 10.1371/journal.pone.0026915

**Published:** 2011-10-28

**Authors:** Mario D. Cordero, Elísabet Alcocer-Gómez, Francisco J. Cano-García, Manuel De Miguel, Angel M. Carrión, Plácido Navas, José A. Sánchez Alcázar

**Affiliations:** 1 Centro Andaluz de Biología del Desarrollo (CABD), Universidad Pablo de Olavide-CSIC and Centro de Investigación Biomédica en Red de Enfermedades Raras (CIBERER), ISCIII, Sevilla, Spain; 2 Departamento Citología e Histología Normal y Patológica, Facultad de Medicina. Universidad de Sevilla, Sevilla, Spain; 3 Departamento de Personalidad, Evaluación y Tratamiento Psicológicos, Facultad de Psicología Universidad de Sevilla, Sevilla, Spain; 4 División de Neurociencias, Universidad Pablo de Olavide de Sevilla, Carretera de Utrera Km. 1, Sevilla, Spain; Hospital Vall d′Hebron, Spain

## Abstract

**Background:**

We examined lipid peroxidation (LPO) in blood mononuclear cells (BMCs) and plasma, as a marker of oxidative damage, and its association to clinical symptoms in Fibromyalgia (FM) patients.

**Methods:**

We conducted a case–control and correlational study comparing 65 patients and 45 healthy controls. Clinical parameters were evaluated using the Fibromyalgia Impact Questionnaire (FIQ), visual analogues scales (VAS), and the Beck Depression Inventory (BDI). Oxidative stress was determined by measuring LPO in BMCs and plasma.

**Results:**

We found increased LPO levels in BMCs and plasma from FM patients as compared to normal control (P<0.001). A significant correlation between LPO in BMCs and clinical parameters was observed (r = 0.584, P<0.001 for VAS; r = 0.823, P<0.001 for FIQ total score; and r = 0.875, P<0.01 for depression in the BDI). We also found a positive correlation between LPO in plasma and clinical symptoms (r = 0.452, P<0.001 for VAS; r = 0.578, P<0.001 for FIQ total score; and r = 0.579, P<0.001 for depression in the BDI). Partial correlation analysis controlling for age and BMI, and sex, showed that both LPO in cells and plasma were independently associated to clinical symptoms. However, LPO in cells, but not LPO in plasma, was independently associated to clinical symptoms when controlling for depression (BDI scores).

**Discussion:**

The results of this study suggest a role for oxidative stress in the pathophysiology of fibromyalgia and that LPO in BMCs rather than LPO in plasma is better associated to clinical symptoms in FM.

## Introduction

Fibromyalgia (FM) is a common chronic pain syndrome with an unknown etiology, which has been associated to a wide spectrum of symptoms like allodynia, debilitating fatigue, joint stiffness and depression. It is diagnosed according to the classification criteria established by the American College of Rheumatology (ACR) [1]. Despite being a common disorder that affects at least 5 million individuals in the United States [2], its pathogenic mechanism remains elusive. Recently, oxidative stress has been proposed as a relevant event in the pathogenesis of this disorder [3]–[6]. Previously, our group has detected decreased coenzyme Q_10_ (CoQ_10_) levels and increased mitochondrial reactive oxygen species (ROS) production in blood mononuclear cells (BMCs) from FM patients [7], [8]. In addition, we have observed that CoQ_10_ and α-tocopherol, two lipophilic antioxidants, induced a significant reduction of ROS in BMCs from FM patients. Taken together, these results suggest that ROS are produced in the lipophilic environment of mitochondrial membranes and that CoQ_10_ deficiency may be involved in oxidative stress in FM [7]. One of the consequences of ROS overproduction is lipid peroxidation (LPO) leading to oxidative destruction of polyunsaturated fatty acids constitutive of cellular membranes and the production of toxic and reactive aldehyde metabolites such as malondialdehyde (MDA) and 4-hydroxynonenal (HNE) [9], [10]. These highly cytotoxic metabolites, produced in relatively large amounts, can diffuse from their site of origin to attack distant targets and form covalent bonds with various molecules [11]–[13]. Therefore, recognition of lipid peroxidation is of interest, as the deleterious effects of this process might be prevented by administration of scavenging systems or antioxidants. MDA assay is one of the most popular methods for assaying LPO in plasma, serum or cell lysates.

Interestingly, there are discrepancies about the correlation between symptoms and LPO and oxidative stress in FM. Significant correlation has been observed between antioxidants levels in plasma and serum, visual analogue scale (VAS) of pain, and morning stiffness [3], [6]. However, Bagis et al. found no correlation between VAS of pain and LPO or superoxide dismutase (SOD) in serum [4]. On the other hand, Ozgocmen et al. found a significant correlation between depression and LPO in serum but not between the biochemical parameters and clinical measures of pain and fatigue [14]. We propose that this controversy could be ascribed to a methodological problem because LPO levels may show higher levels and reflect better the degree of oxidative stress if LPO measurement is performed in cells rather than in plasma or serum. This hypothesis is supported by previous investigations suggesting that mitochondria were the source of ROS in FM [15], [16], and therefore, LPO levels in cells can show better the severity of oxidative stress. Furthermore LPO levels in plasma can be affected by the rate of detoxification by others tissues. Consequently, important information may lack when MDA is measured only in plasma or serum. Therefore we examined the hypothesis that LPO levels in BMCs may be a better oxidative marker than LPO levels in plasma to correlate more significantly and independently with the clinical symptoms in FM patients.

## Methods

### Ethics Statement

Informed consent written and the approval of the ethical committee of University Pablo de Olavide and Universitary Hospital Virgen Macarena from Seville were obtained.

### Patients

In brief, 100 patients from the register of the Sevillian Fibromyalgia Association (AFIBROSE) and 45 healthy matched controls were enrolled into our study. Informed consent and the approval of the local ethical committee were obtained. The inclusion criterion during this study was: Patients diagnosed of FM in the last 2–3 years, based on the current ACR diagnostic criteria [1]. Exclusion criteria were acute infectious diseases in the previous 3 weeks; past or present neurological, psychiatric, metabolic, autoimmune, allergy-related, dermal or chronic inflammatory disease; undesired habits (e.g., smoking, alcohol, etc.); medical conditions that required glucocorticoid treatment, use of analgesics, antidepressants drugs; past or current substance abuse or dependence; and pregnancy or current breastfeeding. Sixty-five potential participants met the inclusion criteria and were enrolled into the study (males/5, females/60), and 35 patients were excluded: 15 were smoker, 13 were using antidepressant treatment, 5 had rheumatoid arthritis, and 2 had hepatitis c. Forty-five healthy volunteers (males/5, females/40) were included in the study matching the age range, gender, ethnicity, and demographics (completion of at least 9 years of education and part of the middle socioeconomic class) of the recruited female FM subjects. Healthy controls had no signs or symptoms of FM and were free of any medication for at least 3 weeks before the study began. All patients and controls had not taken any drug or vitamin/nutritional supplement during a 3 weeks period before the collection of the blood samples. All patients and controls reported followed a standard balanced diet (carbohydrate 50–60%, protein 10–20% and fat 20–30%) that was established by a diet program during 3 weeks before blood collection. The diagnosis of FM was established by an experienced rheumatologist according to ACR criteria [1]. Clinical data were obtained from physical examination, and they were evaluated using the Fibromyalgia Impact Questionnaire (FIQ) including visual analogues scales, and depression with the Beck Depression Inventory (BDI). Tender points were identified by digit pressure of the 18 locations recommended by ACR which included a minimum of 11 out of 18. Heparinized and coagulated blood samples were collected after 12-hours fasting from patients and controls, centrifuged at 3800 g for 5 min, and plasma and serum were stored at −80°C until testing. Serum biochemical parameters were assayed by routine analytical methods. Clinical data and blood samples were collected in a time-frame of 2 months (September and October, 2009).

### Isolation of BMCs

BMCs were purified from heparinized blood by isopycnic centrifugation using Histopaque-1119 and Histopaque-1077 (Sigma Chemical Co., St. Louis, MO, USA).

### Lipid peroxidation

Thiobarbituric acid reactive substances (TBARS) levels in plasma and cells were determined by a method based on the reaction with thiobarbituric acid (TBA) at 90–100°C using a commercial kit from Cayman Chemical Company (Ann Arbor, MI). TBARS are expressed in terms of malondialdehyde (MDA) levels. In these assays, a MDA standard is used to construct a standard curve against which unknown samples can be plotted.

### Statistical analysis

All results are expressed as means ±SD unless stated otherwise. The unpaired Student's t test was used to evaluate the significance of differences between groups. For correlation between LPO levels in BMCs or plasma with clinical parameters, Pearsońn correlation coefficient (r) was performed. Partial correlations for controlling for confounders (BDI, age and BMI, and sex) were also performed. The level of significance was set at p<0,05. As an indicator of internal consistency reliability of the clinical questionnaires, we calculated Cronbachs alpha values (achievable values range from 0, indicating no internal consistency, to 1, indicating identical results). Cronbach's alpha coefficient was 0.795 for FIQ and 0.812 for BDI. Cronbachs alpha values of more than 0.7 are commonly considered markers of a high degree of reliability.

## Results

Mean age in the FM group was 45.8±11 years and in the control group was 44.3±12. Patient routine laboratory tests yield normal results for glucose 90.05±15.16 mg/dL (normal values, n.v. 76–110) , urea 31.02±11.35 mg/dL (n.v. 10–45), uric acid 4.05±1.51 mg/dL (n.v.2.5–7.5), total protein 7.02±0.90 g/dL (n.v. 6.6–8.7), creatinine 0.87±1.11 mg/dL (n.v. 0.5–1.1), aspartate aminotransferase 23.54±5.03 mU/mL (n.v. 10–40), alanine aminotransferase 22.21±10.33 mU/mL (n.v. 10–40), cholesterol 217±36.17 mg/dL (n.v.<220), and triglycerides 99.13±43.5 mg/dL (n.v. 150–200).

**Figure 1:**
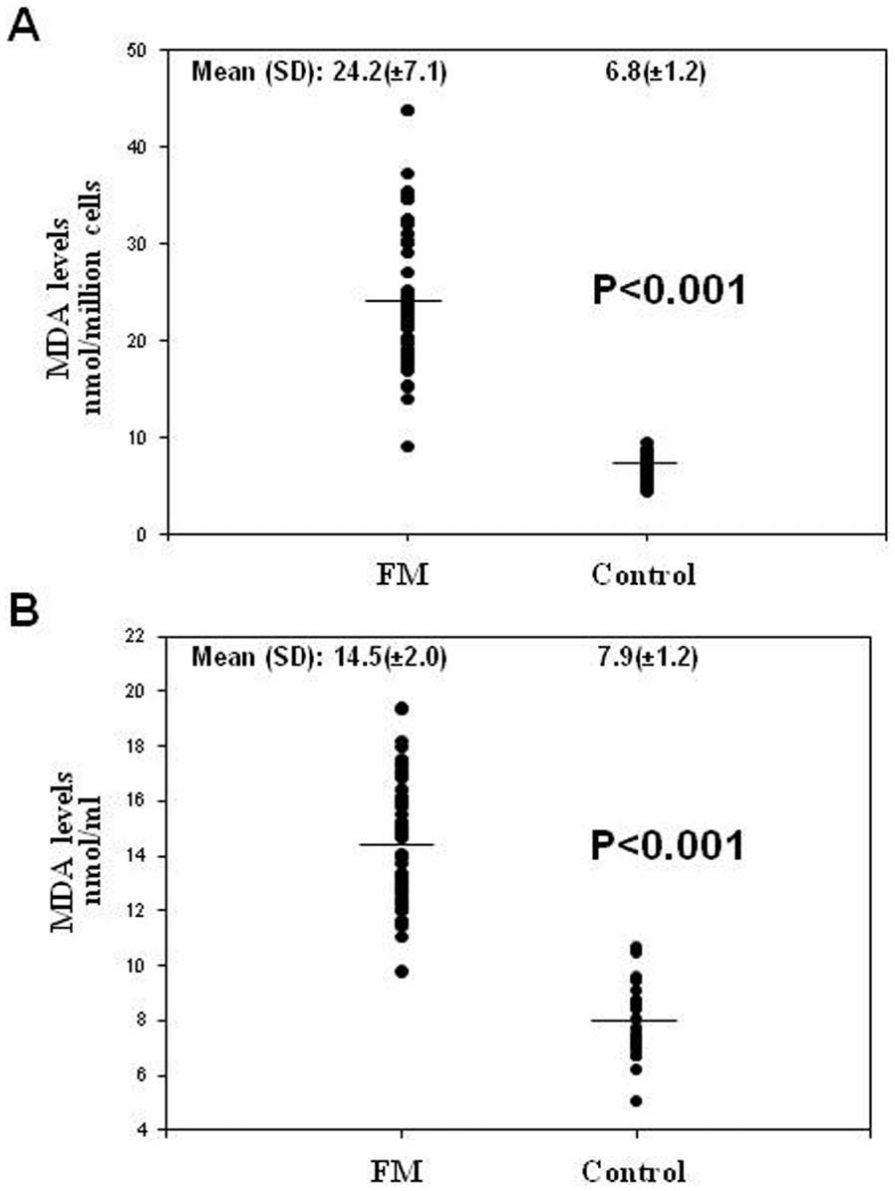
Lipid peroxidation (MDA levels) in BMCs (Panel A) and plasma (Panel B) from controls and FM patients was determined as described in *Material and Methods*. Data represent the mean±SD of three separate experiments. *P<0,001 between control and FM patients.

We determined LPO, as a marker of oxidative stress-induced membrane damage by mitochondrial ROS, in BMCs and plasma from controls and FM patients. On average, FM patients showed higher levels of LPO both in BMCs and plasma respect to controls (p<0.001) (Figure 1A and 1B). Interestingly, LPO levels were strikingly higher in cells (24.2 nmol/million cells = 48.4 nmol/ml of cells lysate) than in plasma (14.17 nmol/ml of plasma) in FM patients (p<0.001). Moreover, both parameters LPO levels in BMCs and LPO levels in plasma were significantly associated. (r = 0.618, P<0.001).

Table 1 shows the clinical characteristics of patients and controls. The mean duration of symptoms in the FM group was 10.4±6.4 years. The mean tender points in the FM group were 15.4±2.8 points (Table 1). The most prominent features of FM patients were pain, fatigue, morning tiredness, and depression (Table 1). As expected, FM patients had markedly higher levels of pain (VAS, 5.9±1.8), depression (BDI, 18.5±8.5), and high overall impact of FM (FIQ, 54.5±16) than in normal controls (P<0.001).

**Table 1:** Characteristic findings of the FM patients and control group.

	Patients		Control	
Age (years)	45.8	±11	44.3	±12
Tender points	15.4	±2.8	?	
Duration of disease (years)	10.4	±6.4	?	
Sex (male/female)	5/60		5/40	
BMI (Kg/m^2^)	27.4	± 4.2	23.3	± 0.9
VAS Total score	5.9	±1.8*	0.5	± 0.8
FIQ Total score, range 0 80	54.5	±16.*	3	±1.6
Pain	7.3	±2.2*	0.7	±0.3
Fatigue	7.6	± 1.9*	1.2	± 0.9
Morning tiredness	6.7	±2.2*	1.1	±1.0
Stiffness	5.9	±2.3*	0.6	±0.1
Anxiety	5.8	±2.7*	1	±0.9
Depression	5.2	±2.7*	1.2	±0.8
Beck Depression Inventory	18.5	±8.6*	4	± 1.9

Values are means ±SD.

*P<0.001.

To study the association between LPO levels in BMCs or plasma and clinical symptoms Pearsońs correlation coefficient (r) was performed. Table 2 shows a significant correlation between LPO levels in BMCs and clinical parameters (r = 0.584, P<0.01 for VAS; r = 0.823, P<0.01 for FIQ total score; r = 0.564, P<0.01 for pain; r = 0.617, P<0.01 for fatigue; r = 0.574, P<0. 01 for morning tiredness; r = 0.669, P<0.01 for stiffness; r = 0.591, P<0.01 for anxiety; r = 0.632, P<0.01 for depression in the FIQ; r = 0.875, P<0.01 for depression in the BDI).

**Table 2:** Correlation between lipid peroxidation (LP) and clinical findings in FM patients.

	Patientsr	Controlr
VAS Total score	0.584**	0.452**
FIQ Total score	0.823**	0578**
Pain	0.564**	0.410**
Fatigue	0.617**	0.311*
Morning tiredness	0.574**	0.397**
Stiffness	0.669**	0.402**
Anxiety	0.591**	0.433**
Depression	0.632**	0.561**
Beck Depression Inventory	0.875**	0.579**

*P<0.05.

**P<0.01; r, Pearson's Correlation Coefficient.

LPO levels in plasma were also significantly associated to clinical parameters (r = 0.452, P<0.01 for VAS; r = 0.578, P<0.01 for FIQ total score; r = 0.410, P<0.01 for pain; r = 0.311, P<0.05 for fatigue; r = 0.397, P<0.01 for morning tiredness; r = 0.402, P<0.01 for stiffness; r = 0.433, P<0.01 for anxiety; r = 0.561, P<0. 01 for depression in the FIQ; r = 0.579, P<0.01 for depression in the BDI).

Interestingly, correlations were higher for both LPO levels in BMCs and plasma with depression than with other clinical parameters.

Next, partial correlation analysis was used to determine the strength of the association of LPO levels in cells or plasma with clinical symptoms, after controlling for depression (BDI), age and BMI, and sex, respectively (Table 3). Partial correlation analysis showed that LPO levels in cells were independently correlated with clinical symptoms, indicating that this significant association was not mediated by depression, age and BMI or sex (Table 3). However, it is remarkable that this association was reduced when controlling for depression (Table 2 and 3). LPO levels in plasma were also independently associated to clinical parameter when controlling for age and BMI and sex. However, the association of LPO levels in plasma and clinical symptoms was eliminated when depression was controlled.

**Table 3:** Partial correlations between lipid peroxidation (LP) and clinical findings controlling for depression (BDI), age and BMI, and sex.

	Beck		Age & BMI		Sex	
	LP Cell r	LP Plasma r	LP Cell r	LP Plasma r	LP Cell r	LP Plasma r
VAS	0.264*	0.200	0.587**	0.459**	0.616**	0.481**
FIQ Total score	0.428**	0.236	0.829***	0.564**	0.824***	0.574**
Pain	0.344**	0.186	0.561**	0.398**	0.577**	0.421**
Fatigue	0.261*	0.045	0.622**	0.291*	0.624**	0.317*
Morning tiredness	0.305*	0.145	0.575**	0.368*	0.569**	0.390**
Stiffness	0.364**	0.080	0.669**	0.385**	0.666**	0.392**
Anxiety	0.283*	0.172	0.600**	0.405**	0.599**	0.440**
Depression	0.155	0.244	0.632**	0.547**	0.644**	0.573**
Beck Depression Inventory	-	-	0.874***	0.576**	0.875***	0.574**

*P<0.05.

**P<0.005.

***P<0.001; r, Pearson's Correlation Coefficient.

## Discussion

The essential findings of this study are that FM patients have significantly elevated levels of LPO in BMCs and plasma (MDA measured as TBARS). Moreover our results show that LPO levels in BMCs are more strongly and independently associated than LPO levels in plasma with FM symptoms. The results presented suggest the involvement of oxidative stress as part of the pathophysiology of FM.

A large number of studies have shown high levels of oxidative stress markers, such as LPO levels, in FM patients, suggesting that this process may have a role in the pathophysiology of this disease. Additionally, we have also shown that BMCs can be an excellent model to study the relation between oxidative stress and FM. [8], [15].

Our results show that FM patients present increased LPO levels in both BMCs and plasma compared to healthy controls, a finding in line with studies by other groups investigating oxidative stress markers in FM [4], [15]. Furthermore, the patients show high levels of pain, fatigue, stiffness, depression, and a high overall impact of FM.

But, what is the relationship between LPO and FM symptoms? It is known that LPO, as a consequence of oxidative stress, indirectly reflects intracellular ROS generation, and ROS are known to be implicated in the etiology of pain, one of the most prominent symptoms in FM, by inducing peripheral and central hyperalgesia [17]. Superoxide plays a major role in the development of pain through direct peripheral sensitization, the release of various cytokines (for example, TNF-α, IL-1β, and IL-6), the formation of peroxynitrite (ONOO-), and poly (ADP-ribose) polymerase activation (PARP) [17]. Although, the mechanisms by which increased oxidative stress can affect specifically muscle sensitivity remain to be established, it may be that oxidative damage in muscles results in threshold lowering of nociceptors locally, thus producing and altered nociception [18].

Furthermore, increased LPO has been described in patients suffering from depression and fatigue, two typical symptoms found in FM patients [19], [20]. Studies on depression have elucidated a possible link between depression and LPO [21], and the peroxidation-reducing effect of different selective serotonin reuptake inhibitors in major depression has been demonstrated by Bilici et al. [19]. It has been suggested that alterations in phospholipids which are structural components of cell membrane in the brain, may induce changes in membrane microviscosity and, consequently, in various neurotransmitter systems, which are thought be related to the pathology of depression, e.g., serotonin (5-HT), and noradrenaline [22], [23]. LPO of cell membranes can modify receptor accessibility, dynamics, ligand binding and action, and therefore altering neurotransmitter functions [24]. Oxidative stress may also affect the expression of membrane functional proteins and receptors, by interfering with intracellular signalling and receptors turnover, including serotonergic receptors [25].

The fact that depression was associated to both LPO in BMCs and plasma can be ascribed to the fact that neuron membranes can be more vulnerable to oxidative stress due to its high content in polyunsaturated fatty acids [23], and consequently neurotransmitters pathways involved in depression may be more easily affected.

Furthermore, this association was higher with LPO in BMCs than in plasma in all the clinical parameters analyzed. These findings could be ascribed to the presence of higher levels of LPO in BMCs than in plasma that can reflect better the severity of oxidative stress, and thus, showing a better association to clinical symptoms in FM. MDA is a end-product of LPO, and its cellular accumulation indicates oxidative damage in the cells. However, plasma MDA levels depend on the balance between MDA formation and its detoxification and can be affected for many factors, such as the dilutional effect of plasma, and the renal and/or tissue clearance. The fact that severity of FM symptoms apparently correspond better with BMCs MDA values than plasma MDA values, might be also speculated as a predominant contribution of the inflammatory cells in the severity of FM symptoms.

Furthermore, depression *per se* is accompanied by an induction of inflammatory and oxidative and nitrosative stress pathways, altered cytokine activity [26], increased LPO levels and a lowered antioxidant status [27]–[29]. All these alterations, which may reinforce themselves through a feedback mechanism (depression induces LPO and LPO induces depression), make a significant a much stronger case for the association between LPO levels in cells or plasma and depression in FM patients.

The inflammation, oxidative and nitrosative stress theory of depression offers an explanation why depression may be caused by psychological stressors and the presence of (auto)immune disorders [28]. The latter is indeed accompanied by inflammatory responses that may induce oxidative and nitrosative stress whereby NO and peroxynitrite are formed by for example activated neutrophils and monocytes, which in turn may cause oxidation and nitration of fatty acids, proteins and DNA. Psychological stressors may generate oxidative stress and even cause damage to fatty acids and DNA. Firstly, emotional stressors induce inflammatory reactions with an increased production of pro-inflammatory cytokines [30], which cause ROS and reactive nitrogen species (RNS). Secondly, psychological stressors induce a pro-oxidant state and LPO [31], [32]. Therefore, the high comorbidity between FM and depression may be explained because both share overlapping pathophysiological processes [33], [34].

Furthermore, we examined the independence of LPO levels and clinical parameters association in FM and the modulating role of depression (BDI) and others confounders (age and BMI, and sex). Partial correlation analysis showed that LPO levels in BMCs correlated with clinical symptoms independently of depression, age and BMI, and sex in FM patients. Interestingly, this association was reduced when controlling for depression, suggesting that in some extend depression is modulating LPO levels in BMCs. On the contrary, LPO levels in plasma were not associated with clinical parameters when controlling for depression, suggesting that depression has a confounding effect on LPO levels in plasma in FM patients.

In conclusion, our study confirms the presence of LPO in BMCs and plasma in FM patients. LPO levels in BMCs are better associated than LPO levels in plasma to clinical symptoms in FM. Furthermore, this association, although significantly independent, is modulated by depression. Finally, the results of this study indicate that oxidative stress could be implicated in the severity of the clinical symptoms in FM and suggest that antioxidant therapy needs to be examined as a treatment in FM. Recently, The role of oxidative stress in peripheral neuropathic pain was recently tested by assessing the effects of antioxidants (acetyl-L-carnitine, alpha-lipoic acid, and vitamin C) on pain behavior in a rat model of neuropathic pain [32]. Our results might also be relevant for the clinical management of FM patients since the effectiveness of treatment could be additionally tested by serial BMCs LPO determinations during the course of the treatment.

## References

[1] Wolfe F, Smythe HA, Yunus MB, Bennett RM, Bombardier C (1990). The American College of Rheumatology 1990 Criteria for the Classification of Fibromyalgia. Report of the Multicenter Criteria Committee.. Arthritis Rheum.

[2] Lawrence RC, Felson DT, Helmick CG, Arnold LM, Choi H (2008). Estimates of the prevalence of arthritis and other rheumatic conditions in the United States. Part II.. Arthritis Rheum.

[3] Altindag O, Celik H (2006). Total antioxidant capacity and the severity of the pain in patients with fibromyalgia.. Redox Rep.

[4] Bagis S, Tamer L, Sahin G, Bilgin R, Guler H (2005). Free radicals and antioxidants in primary fibromyalgia: an oxidative stress disorder?. Rheumatol Int.

[5] Ozgocmen S, Ozyurt H, Sogut S, Akyol O (2006). Current concepts in the pathophysiology of fibromyalgia: the potential role of oxidative stress and nitric oxide.. Rheumatol Int.

[6] Sendur OF, Turan Y, Tastaban E, Yenisey C, Serter M (2009). Serum antioxidants and nitric oxide levels in fibromyalgia: a controlled study.. Rheumatol Int.

[7] Cordero MD, de Miguel M, Moreno-Fernandez AM (2010). [Mitochondrial dysfunction in fibromyalgia and its implication in the pathogenesis of disease.].. Med Clin (Barc).

[8] Cordero MD, Moreno-Fernandez AM, deMiguel M, Bonal P, Campa F (2009). Coenzyme Q10 distribution in blood is altered in patients with fibromyalgia.. Clin Biochem.

[9] Draper HH, Csallany AS, Hadley M (2000). Urinary aldehydes as indicators of lipid peroxidation in vivo.. Free Radic Biol Med.

[10] Esterbauer H, Schaur RJ, Zollner H (1991). Chemistry and biochemistry of 4-hydroxynonenal, malonaldehyde and related aldehydes.. Free Radic Biol Med.

[11] Benedetti A, Casini AF, Ferrali M, Comporti M (1979). Effects of diffusible products of peroxidation of rat liver microsomal lipids.. Biochem J.

[12] Clot P, Tabone M, Arico S, Albano E (1994). Monitoring oxidative damage in patients with liver cirrhosis and different daily alcohol intake.. Gut.

[13] Niemela O, Parkkila S, Yla-Herttuala S, Halsted C, Witztum JL (1994). Covalent protein adducts in the liver as a result of ethanol metabolism and lipid peroxidation.. Lab Invest.

[14] Ozgocmen S, Ozyurt H, Sogut S, Akyol O, Ardicoglu O (2006). Antioxidant status, lipid peroxidation and nitric oxide in fibromyalgia: etiologic and therapeutic concerns.. Rheumatol Int.

[15] Cordero MD, De Miguel M, Moreno Fernandez AM, Carmona Lopez IM, Garrido Maraver J (2010). Mitochondrial dysfunction and mitophagy activation in blood mononuclear cells of fibromyalgia patients: implications in the pathogenesis of the disease.. Arthritis Res Ther.

[16] Cordero MD, Miguel MD, Carmona-Lopez I, Bonal P, Campa F (2010). Oxidative stress and mitochondrial dysfunction in Fibromyalgia. MINIREVIEW.. Neuro Endocrinol Lett.

[17] Wang ZQ, Porreca F, Cuzzocrea S, Galen K, Lightfoot R (2004). A newly identified role for superoxide in inflammatory pain.. J Pharmacol Exp Ther.

[18] Fulle S, Mecocci P, Fano G, Vecchiet I, Vecchini A (2000). Specific oxidative alterations in vastus lateralis muscle of patients with the diagnosis of chronic fatigue syndrome.. Free Radic Biol Med.

[19] Bilici M, Efe H, Koroglu MA, Uydu HA, Bekaroglu M (2001). Antioxidative enzyme activities and lipid peroxidation in major depression: alterations by antidepressant treatments.. J Affect Disord.

[20] Vecchiet J, Cipollone F, Falasca K, Mezzetti A, Pizzigallo E (2003). Relationship between musculoskeletal symptoms and blood markers of oxidative stress in patients with chronic fatigue syndrome.. Neurosci Lett.

[21] Evans PH (1993). Free radicals in brain metabolism and pathology.. Br Med Bull.

[22] Maes M, Smith R, Christophe A, Cosyns P, Desnyder R (1996). Fatty acid composition in major depression: decreased omega 3 fractions in cholesteryl esters and increased C20: 4 omega 6/C20:5 omega 3 ratio in cholesteryl esters and phospholipids.. J Affect Disord.

[23] Tsutsumi S, Tsuji K, Ogawa K, Ito T, Satake T (1988). Effect of dietary salt and cholesterol loading on vascular adrenergic receptors.. Blood Vessels.

[24] Lenaz G (1987). Lipid fluidity and membrane protein dynamics.. Biosci Rep.

[25] Maes M, Mihaylova I, Leunis JC (2007). Increased serum IgM antibodies directed against phosphatidyl inositol (Pi) in chronic fatigue syndrome (CFS) and major depression: evidence that an IgM-mediated immune response against Pi is one factor underpinning the comorbidity between both CFS and depression.. Neuro Endocrinol Lett.

[26] Licinio J, Wong ML (1999). The role of inflammatory mediators in the biology of major depression: central nervous system cytokines modulate the biological substrate of depressive symptoms, regulate stress-responsive systems, and contribute to neurotoxicity and neuroprotection.. Mol Psychiatry.

[27] Galecki P, Szemraj J, Bienkiewicz M, Florkowski A, Galecka E (2009). Lipid peroxidation and antioxidant protection in patients during acute depressive episodes and in remission after fluoxetine treatment.. Pharmacol Rep.

[28] Maes M (2008). The cytokine hypothesis of depression: inflammation, oxidative & nitrosative stress (IO&NS) and leaky gut as new targets for adjunctive treatments in depression.. Neuro Endocrinol Lett.

[29] Maes M, Mihaylova I, Kubera M, Uytterhoeven M, Vrydags N (2009). Lower plasma Coenzyme Q10 in depression: a marker for treatment resistance and chronic fatigue in depression and a risk factor to cardiovascular disorder in that illness.. Neuro Endocrinol Lett.

[30] Maes M, Song C, Lin A, De Jongh R, Van Gastel A (1998). The effects of psychological stress on humans: increased production of pro-inflammatory cytokines and a Th1-like response in stress-induced anxiety.. Cytokine.

[31] Pertsov SS, Balashova TS, Kubatieva AA, Sosnovskii AS, Pirogova GV (1995). [Lipid peroxidation and antioxidant enzymes in rat brain in acute emotional stress: effect of interleukin-1beta].. Biull Eksp Biol Med.

[32] Sosnovskii AS, Kozlov AV (1992). [Increased lipid peroxidation in the rat hypothalamus after short-term emotional stress].. Biull Eksp Biol Med.

[33] Aguglia A, Salvi V, Maina G, Rossetto I, Aguglia E (2011). Fibromyalgia syndrome and depressive symptoms: Comorbidity and clinical correlates.. J Affect Disord.

[34] Maletic V, Raison CL (2009). Neurobiology of depression, fibromyalgia and neuropathic pain.. Front Biosci.

